# Impact of Oral Hygiene on Prognosis in Patients With Squamous Cell Carcinoma of the Lower Gingiva

**DOI:** 10.3389/fsurg.2021.711986

**Published:** 2021-09-21

**Authors:** Hui Zhao, Shengnan Zhang, Jinji Ma, Xiaodong Sun

**Affiliations:** ^1^Department of Endodontics of East Branch of Jinan Stomatological Hospital, Jinan, China; ^2^Department of Prosthodontics of East Branch of Jinan Stomatological Hospital, Jinan, China; ^3^Department of Orthodontics of Gaoxin Branch of Jinan Stomatological Hospital, Jinan, China; ^4^Department of Endodontics of Gaoxin Branch of Jinan Stomatological Hospital, Jinan, China

**Keywords:** oral squamous cell carcinoma, oral hygiene, survival significance, prognosis, lower gingiva SCC

## Abstract

**Objective:** We aimed to analyze the significance of oral hygiene in patients with squamous cell carcinoma of the lower gingiva.

**Methods:** Oral hygiene was assessed using a questionnaire by calculating the oral health (OH) score and the dental care (DC) score. The association of oral hygiene with clinical pathologic variables, disease free survival (DFS), and overall survival (OS) was analyzed.

**Results:** Four out of 53 non-smokers and 19 out of 90 current or former smokers had an OH score of 6 (statistically significant difference, *p* = 0.036). Fifteen out of 63 patients with a T3/T4 tumor and 8 out of 80 patients with a T1/T2 tumor had an OH score of 6 (statistically significant difference, *p* = 0.026). Similar statistically significant findings were noted with respect to the DC scores, smoking status, and tumor stage. Among patients with an OH score of 0–5, the 5-year DFS and OS rates were 55 and 50%, respectively, and among patients with an OH score >5, they were 46 and 43%, respectively (both differences statistically significant, *p* < 0.05). Among patients with a DC score of 0–2, the 5-year DFS and OS rates were 69 and 51%, respectively and among patients with a DC score >2, they were 50 and 47%, respectively (both differences statistically significant, *p* < 0.05). Cox model confirmed OH and DC scores as independent factors affecting the DFS and OS.

**Conclusion:** Poor oral hygiene was associated with decreased DFS and OS.

## Introduction

Head and neck squamous cell carcinoma (SCC) is the seventh most common malignancy in the world (1). The common risk factors include smoking, drinking, betel nut chewing, and human papillomavirus infection (2, 3). Additionally, current evidence strongly suggests that low frequency of tooth brushing, tooth loss, need or current use of a prosthesis, and non-regular visits to the dentist are associated with the development of head and neck SCC (4–7). All these factors are directly or indirectly related to periodontitis. Moreover, a few authors have suggested that self-reported oral health indicators including frequency of routine dental examinations and frequency of tooth brushing significantly affect the survival in head and neck SCC (8, 9).

Since gingiva is an important part of periodontal tissues, initial symptoms of stomatitis are usually related to the gingiva. Hence, it is speculated that there might be a unique effect of oral hygiene on SCC of gingiva. However, this topic has never been analyzed in detail. Therefore, we aimed to assess the significance of oral health in patients with SCC of lower gingiva.

## Patients and Methods

### Ethics

The institutional research committee of our hospital approved this study and all participants signed an informed consent agreement. All procedures were conducted in accordance with the ethical standards of the institutional and/or national research committee and the 1964 Declaration of Helsinki and its later amendments or comparable ethical standards.

### Patient Selection

Medical records of patients with surgically treated SCC of lower gingiva were retrospectively reviewed between January 2014 and December 2020. The inclusion criteria were primary disease, no history of other cancers, and adequate follow-up data. Information regarding demography, Eastern Cooperative Oncology Group (ECOG) performance status, pathology, treatment, and follow-up of enrolled patients was extracted.

### Oral Hygiene Assessment

A questionnaire evaluating the oral hygiene was sent to the patients and/or their family via email, WeChat, or post. The questionnaire was constructed based on repeatedly reported variables associated with head and neck SCC (6, 9–11). The questionnaire consisted of two items: the oral health (OH) score and the dental care (DC) score (Table 1). The OH score was based on three indicators, namely wearing of dentures, age at the start of wearing dentures, and the frequency of gum bleeding while tooth brushing. The DC score was based on three indicators, namely frequency of tooth cleaning; use of a toothbrush, toothpaste, or dental floss; and the frequency of visits to a dentist. Higher composite scores indicated poorer oral hygiene.

**Table 1:** Operationalizing of the composite score.

**Oral health**	**Score**	**Specification**
Wearing of dentures	0	no denture
	1	partial denture in upper or lower jaw
	2	partial denture in both jaws
	3	complete denture in upper or lower jaw
	4	complete denture in both jaws
Age at starting to wear dentures	0	no denture
	1	denture at age 55 or older
	2	denture at age 35–54 years
	3	denture at age below 35 years
Frequency of gum bleeding from brushing teeth	0^*^	sometimes or never
	1	always or almost always
Dental care	Score	Specification
Frequency of teeth cleaning	0	at least twice/day
	1	once/day
	2	1–4 times/week
	3	less often or never
Use of toothbrush, toothpaste or dental floss	0	two or three of these
	1	only one of these three
	2	none of these
Frequency of dentist visits	0	at least once a year
	1	every 2–5 years
	2	less than every 5 years
	3	Never

* *Score 0 was always applied in patients with complete dentures in both jaws*.

### Treatment Proposal

In our cancer center, systemic examinations including ultrasound, computed tomography, and/or magnetic resonance imaging were performed for every patient with any stage of SCC of lower gingiva. Marginal or segmental mandibulectomy with a safe margin of at least 1 cm was performed based on the disease stage and the extent of invasion. Neck dissection was routinely performed with level 1–3 dissection for a cN0 neck and level 1–5 dissection for for a cN+ neck (12). Adjuvant therapies were performed if there was presence of T3/T4 tumor, positive neck lymph nodes, perineural invasion, lymphovascular invasion, positive margin, or extracapsular spread.

### Definitions of Important Variables

Current drinkers were defined as those who consumed at least one alcoholic drink per day for at least 1 year. Non-drinkers were defined as those who consumed an alcoholic drink no more than once every 2 weeks in their lifetime. The remaining patients were defined as former drinkers. Current smokers were defined as those who smoked on a daily basis or had quit smoking <5 years ago. Non-smokers were defined as those who had smoked no more than 100 cigarettes in their lifetime. The remaining patients were defined as former smokers (2, 12–14). Perineural invasion was considered to be present if tumor cells were identified within the perineural space and/or nerve bundle. Lymphovascular invasion was considered positive if tumor cells were noted within the lymphovascular channels. Extracapsular spread was considered positive if tumor cells were observed outside the capsule of a metastatic lymph node.

### Statistical Analysis

Associations between clinicopathologic variables and oral hygiene were evaluated using the chi-squared test. The Kaplan-Meier method was used to assess the disease free survival (DFS) and the overall survival (OS). The DFS was calculated from the date of surgery to the date of disease recurrence or the last follow-up. The OS was calculated from the date of surgery to the date of death or the last follow-up. Factors that were significant in the univariate analyses were subsequently analyzed in a Cox model to determine the independent prognostic factors. All statistical analyses were performed using IBM SPSS Statistics, version 20.0 (IBM Corp., Armonk, NY, USA) and *p* < 0.05 was considered statistically significant.

## Results

### Baseline Data

Altogether, 206 questionnaires were sent to 206 enrolled patients and 143 (69.4%) patients completed the questionnaires. Among these 143 patients, 105 (73.4%) were male and 38 (26.6%) were female, with a mean age of 50.5 (range: 30–75) years. Seventy (49.0%), 20 (14.0%), and 53 (37.1%) patients were current smokers, former smokers, and non-smokers, respectively. Forty (28.0%), 13 (9.1%), and 90 (62.9%) patients were current drinkers, former drinkers, and non-drinkers, respectively. American Society of Anesthesiologists physical status was I in 49 (34.2%) patients, II in 58 (40.6%) patients, and III in 36 (25.2%) patients. The ECOG performance status was 0 in 89 (62.2%) patients and 1 in 54 (37.8%) patients.

Pathological tumor stage was T1 in 24 (16.8%) patients, T2 in 56 (39.2%) patients, T3 in 37 (25.9%) patients, and T4 in 26 (18.2%) patients. The tumor was well-differentiated in 40 (28.0%) patients, moderately differentiated in 57 (40.0%) patients, and poorly differentiated in 46 (32.2%) patients. Perineural invasion and lymphovascular invasion were observed in 36 (25.2%) and 30 (21.0%) patients, respectively. Pathological cervical nodal stage was N0 in 85 (59.4%) patients, N1 in 30 (21.0%) patients, N2 in 18 (12.6%) patients, and N3 in 10 (7.0%) patients. Extracapsular spread was observed in 10 (7.0%) patients. Positive margin was present in 8 (5.6%) patients.

### The OH Score

OH scores of 0, 1, 2, 3, 4, 5, and 6 were noted in 30 (21.0%), 60 (42.0%), 8 (5.6%), 9 (6.3%), 13 (9.1%), and 23 (16.1%) patients, respectively. Four out of 53 non-smokers and 19 out of 90 current or former smokers had an OH score of 6 and the difference was statistically significant (*p* = 0.036). Fifteen out of 63 patients with a T3/T4 tumor and 8 out 80 patients with a T1/T2 tumor had an OH score of 6 and the difference was statistically significant (*p* = 0.026). No significant differences were noted between other variables and the OH score (Table 2).

**Table 2:** Association between oral hygiene and clinical pathologic variables.

**Variable**	**Oral health score**		***P***	**Dental care score**		***p***
	**0–5**	**>5**		**0–2**	**>2**	
	**(*n* = 120)**	**(*n* = 23)**		**(*n* = 28)**	**(*n* = 115)**	
**Age**						
<40	10	3		5	8	
≥40	110	20	0.693	23	107	0.133
**Gender**						
Male	90	15		17	88	
Female	30	8	0.331	11	27	0.089
**ASA**						
I	40	9		9	40	
II	49	9		10	48	
III	31	5	0.847	9	27	0.632
ECOG^*^						
0	75	14		16	73	
1	45	9	0.883	12	42	0.535
**Smoker**						
Current + former	71	19		12	78	
Never	49	4	0.036	16	37	0.014
**Drinker**						
Current + former	43	10		9	44	
Never	77	13	0.487	19	71	0.548
**Tumor differentiation**						
Well	36	4		5	35	
Moderate	47	10		12	45	
Poor	37	9	0.449	11	35	0.387
**Perineural invasion**						
Presence	30	6		8	28	
Absence	90	17	0.912	20	87	0.644
**Lymphovascular invasion**						
Presence	25	5		7	23	
Absence	95	18	1.000	21	92	0.560
**Tumor stage**						
T1+T2	72	8		21	59	
T3+T4	48	15	0.026	7	56	0.024
**Neck nodal stage**						
N0	70	15		17	68	
N+	50	8	0.538	11	47	0.878

* *ECOG, Eastern Cooperative Oncology Group*.

### The DC Score

DC scores of 0, 1, 2, 3, 4, 5, 6, 7, and 8 were noted in 8 (5.6%), 10 (7.0%), 10 (7.0%), 13 (9.1%), 15 (10.5%), 20 (14.0%), 30 (21.0%), and 37 (25.9%) patients, respectively. Thirty-seven out of 53 non-smokers and 78 out of 90 current or former smokers had a DC score of 3 or higher and the difference was statistically significant (*p* = 0.014). Fifty-six out of 63 patients with a T3/T4 tumor and 59 out of 80 patients with a T1/T2 tumor had a DC score of 3 or higher and the difference was statistically significant (*p* = 0.024). No significant differences were noted between other variables and the DC score (Table 2).

### Survival Data

**Figure 1:**
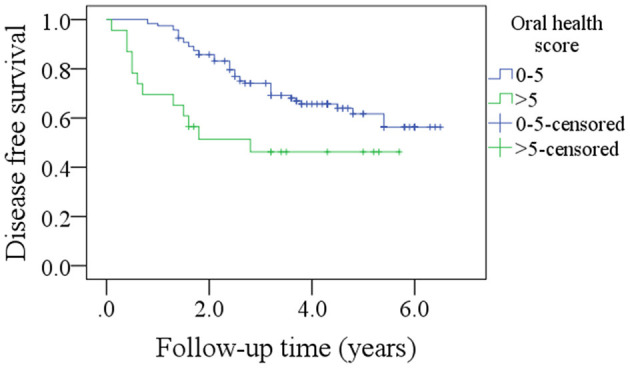
Comparison of disease free survival in patients with different oral health scores (*p* = 0.007).

**Figure 2:**
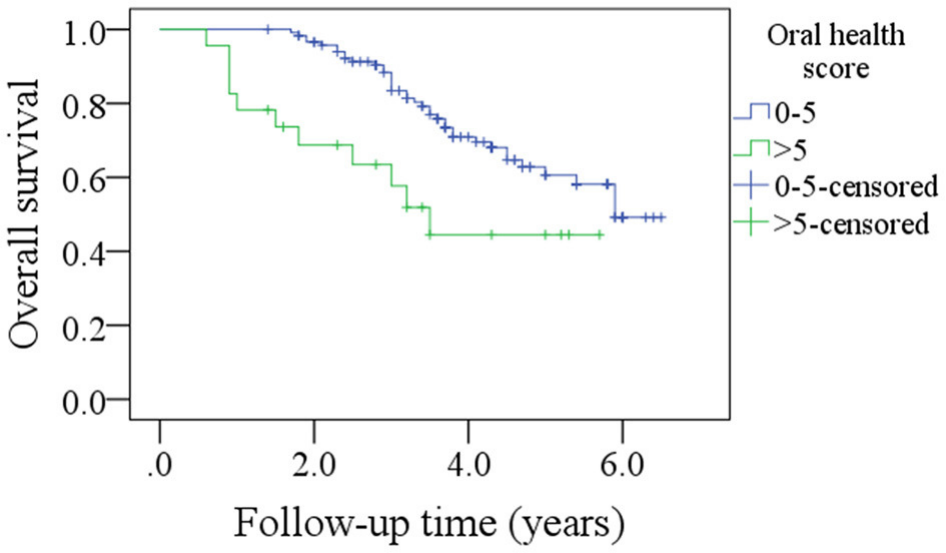
Comparison of overall survival in patients with different oral health scores (*p* = 0.004).

After a median follow-up of 3.6 years, 54 patients developed disease recurrence and 49 patients died. The 5-year DFS and OS rates were 53 and 49%, respectively. Among patients with an OH score of 0–5, the 5-year DFS rate was 55% and among patients with an OH score >5, it was 46%. The difference was statistically significant (Figure 1, *p* = 0.007). Among patients with an OH score of 0–5, the 5-year OS rate was 50% and among patients with an OH score >5, it was 43%. The difference was statistically significant (Figure 2, *p* = 0.004). The Cox model confirmed that the OH score was an independent factor affecting the DFS (Table 3) and OS (Table 4).

**Table 3:** Univariate and Cox model analyses of disease free survival in the 143 patients.

**Variable**	**Univariate**	**Cox model**	
	**Log-rank**	***P***	**HR [95% CI]**
Age (<40 vs. ≥40)	0.345		
Gender	0.473		
ASA (III vs. I+II)	0.221		
ECOG^*^ (1 vs. 0)	0.564		
Smoker (Current+former vs. never)	<0.001	0.081	2.343 [0.926–3.567]
Drinker (Current+former vs. never)	0.098		
Tumor differentiation (poor vs. others)	<0.001	<0.001	4.327 [1.986–9.667]
Perineural invasion	<0.001	0.011	2.567 [1.227–6.433]
Lymphovascular invasion	<0.001	0.233	2.443 [0.832–8.335]
Tumor stage (T3+T4 vs. T1+T2)	<0.001	<0.001	3.658 [1.889–9.337]
Neck nodal stage (N+ vs. N0)	<0.001	<0.001	3.675 [1.674–7.559]
Oral health score (>5 vs. 0–5)	0.007	0.011	2.118 [1.328–4.332]
Dental care score (>2 vs. 0–2)	0.027	0.008	2.333 [1.436–5.321]

* *ECOG, Eastern Cooperative Oncology Group*.

**Table 4:** Univariate and Cox model analyses of overall survival in the 143 patients.

**Variable**	**Univariate**	**Cox model**	
	**Log-rank**	***P***	**HR [95% CI]**
Age (<40 vs. ≥40)	0.276		
Gender	0.438		
ASA (III vs. I+II)	0.387		
ECOG^*^ (1 vs. 0)	0.021	0.075	2.119 [0.975–3.943]
Smoker (Current+former vs. never)	0.004	<0.001	2.663 [1.345–5.432]
Drinker (Current+former vs. never)	0.111		
Tumor differentiation (poor vs. others)	<0.001	<0.001	3.227 [1.874–8.332]
Perineural invasion	0.011	0.211	2.564 [0.823–6.432]
Lymphovascular invasion	0.001	0.108	2.543 [0.763–7.003]
Tumor stage (T3+T4 vs. T1+T2)	<0.001	<0.001	4.398 [2.203–10.443]
Neck nodal stage (N+ vs. N0)	<0.001	<0.001	3.278 [1.874–8.667]
Oral health score (>5 vs. 0–5)	0.004	0.004	1.987 [1.234–3.447]
Dental care score (>2 vs. 0–2)	0.038	0.002	2.001 [1.465–4.221]

* *ECOG, Eastern Cooperative Oncology Group*.

**Figure 3:**
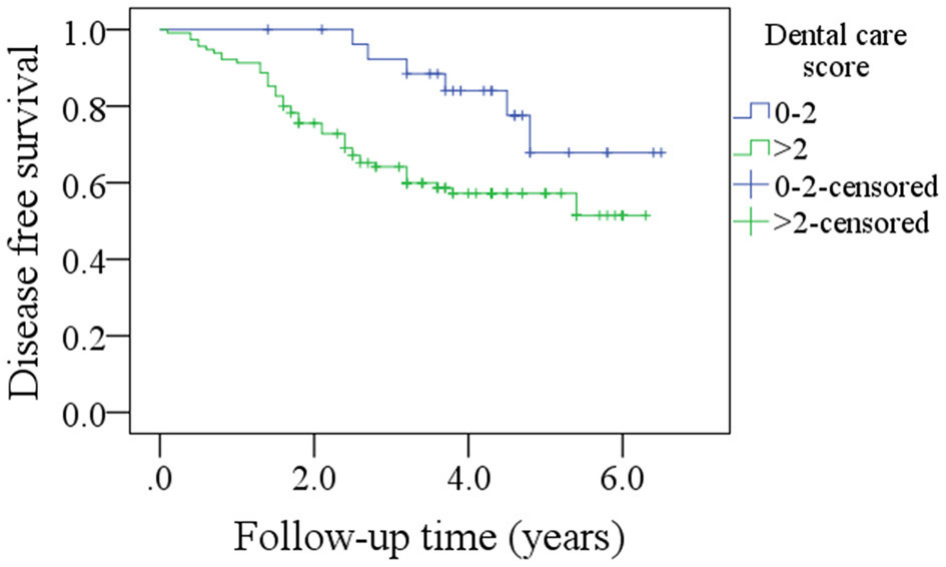
Comparison of disease free survival in patients with different dental care scores (*p* = 0.027).

**Figure 4:**
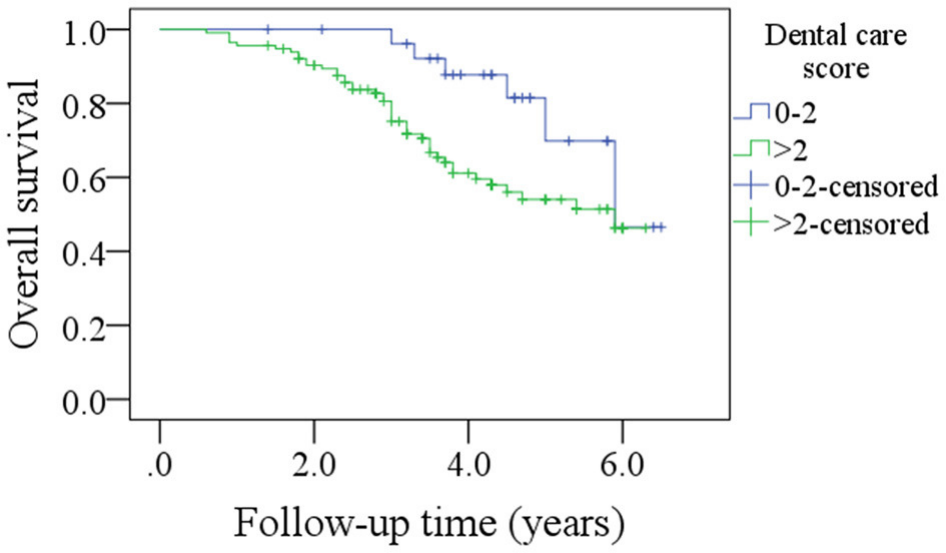
Comparison of overall survival in patients with different oral health scores (*p* = 0.038).

Among patients with a DC score of 0–2, the 5-year DFS rate was 69% and among patients with a DC score of >2, it was 50%. The difference was statistically significant (Figure 3, *p* = 0.027). Among patients with a DC score of 0–2, the 5-year OS rate was 51% and among patients with a DC score >2, it was 47%. The difference was statistically significant (Figure 4, *p* = 0.038). The Cox model confirmed that the DC score was an independent factor affecting the DFS (Table 3) and OS (Table 4).

## Discussion

The most important finding in the present study was that both OH and DC score were significantly associated with smoking and tumor stage. Moreover, high OH and DC scores predicted worse DFS and OS. These findings emphasize the effect of oral hygiene on survival in patients with SCC of lower gingiva and suggest the requirement for more adjuvant treatments in the presence of features related to poor oral health.

Good oral hygiene has been defined as clean teeth, absence of caries, no oral pain, normal gingival color, and no gingival bleeding (15). It consists of at least three aspects, namely good oral health, sound oral function, and no oral disease. Ample evidence has shown that poor oral hygiene status is related to an increased risk for various systemic disorders (16) and solid tumors including head and neck SCCs (4–7). However, very few researchers have analyzed the effect of oral hygiene on the prognosis of patients with head and neck SCC. To the best of our knowledge, only two papers have discussed this issue. Friemel et al. (9) was the first to assess the association between survival duration and oral health behavior. The authors enrolled 276 patients with head and neck SCC and grouped them using weighted composite scores based on self-reported information regarding gum bleeding, wearing of dentures, use of floss, tooth brushing, and visits to a dentist. The authors reported that good dental care scores, summarizing annual dental visits, daily teeth cleaning, and use of floss were associated with longer OS. Similarly, the Cox regression analysis suggested a higher risk of tumor progression and shortened OS in patients with poor dental care. However, the results lost their statistical significance after controlling for other types of health behaviors. Frequent use of mouthwash (≥2 times/day) significantly increased the risk of tumor-specific death. Alcohol consumption and tobacco smoking were associated with tumor progression and shorter overall survival in a dose-dependent manner. A study by Farquhar et al. (8) included 1,381 head and neck SCC patients and 1,396 age, sex, and race-matched controls. Oral health was assessed using self-reported indicators including tooth brushing and the frequency of routine dental examinations. The authors reported that >10 dental visits during the preceding 10 years were associated with decreased risk of mortality after adjusting for confounders in the experimental group. This effect was most pronounced in case of oral cavity cancers. Among controls, dental visits were positively associated with survival. Both the aforementioned studies confirmed the positive relationship between oral health markers and survival in patients with head and neck SCC. More importantly, this association was most pronounced at sites closer to the dentition. Oral health may have a direct effect on tumor biology due to the associated immune or inflammatory responses (8), which are associated with clinical symptoms in the gingiva. In the present study, poor oral hygiene predicted worse DFS and OS in patients with SCC of lower gingiva. Possible explanations might include the following. (1) Oral hygiene is an important part of general body health and they influence each other. Poor oral hygiene might be a local manifestation of a systemic disease. Conversely, it may promote the development of a systemic disease (15, 16). (2) Oral hygiene partially reflects the socioeconomic status. Patients with poor oral hygiene usually belong to low-income families and have poor educational background. Thus, inadequate supporting might contribute to the variation in survival such as tooth loss. Abnet et al. (17) followed 29,584 healthy, rural Chinese adults and categorized tooth loss in each subject as less than/equal to or greater than the median number of teeth lost in other subjects of the same age at baseline. The authors reported that individuals with greater number of lost teeth than the age-specific median number of lost teeth had significantly (13%) greater risk of death. Similar findings were also noted by Goto et al. (18) and Tu et al. (19). (3) Local inflammation induced by poor oral hygiene also plays an important role. The possible mechanisms include induction of chronic inflammation, promotion of cellular invasion, and direct production of carcinogens (20–22).

Interestingly, high OH and DC scores were associated with smoking and higher tumor stage in the present study. Smoke contains numerous types of toxic substances and easily causes inflammatory reaction (23). Smokers usually exhibit poor oral hygiene. Additionally, high OH and DC scores partially suggested poor health behavior such as lack of willingness for regular or timely dental visits. Thus, an early-stage tumor might not be detected until there is presence of pain or bleeding caused by an advanced-stage tumor (24).

Some limitation of the present study must be acknowledged. Oral hygiene was assessed using a subjective questionnaire. Hence, there is a possibility of recall bias. The rate of questionnaire completion was just 69.4%. Hence, the results might not indicate the actual situation. The sample size was relatively small and the statistical power was limited.

In conclusion, smokers with T3/T4 SCC of lower gingiva were likely to exhibit higher OH and DC scores and and poor oral hygiene was associated with decreased DFS and OS.

## Data Availability Statement

The original contributions presented in the study are included in the article/supplementary material, further inquiries can be directed to the corresponding author/s.

## Ethics Statement

The studies involving human participants were reviewed and approved by the institutional research committee of our hospital approved this study and all participants signed an informed consent agreement. The patients/participants provided their written informed consent to participate in this study.

## Author Contributions

All authors listed have made a substantial, direct and intellectual contribution to the work, and approved it for publication.

## Conflict of Interest

The authors declare that the research was conducted in the absence of any commercial or financial relationships that could be construed as a potential conflict of interest.

## Publisher's Note

All claims expressed in this article are solely those of the authors and do not necessarily represent those of their affiliated organizations, or those of the publisher, the editors and the reviewers. Any product that may be evaluated in this article, or claim that may be made by its manufacturer, is not guaranteed or endorsed by the publisher.
